# Influence of Eu^3+^ Doping on Physiochemical Properties and Neuroprotective Potential of Polyacrylic Acid Functionalized Cerium Oxide Nanoparticles

**DOI:** 10.3390/ijms25052501

**Published:** 2024-02-21

**Authors:** Rugmani Meenambal, Tomasz Kruk, Klaudia Jakubowska, Jacek Gurgul, Krzysztof Szczepanowicz, Marta Szczęch, Lilianna Szyk-Warszyńska, Piotr Warszyński, Danuta Jantas

**Affiliations:** 1 Maj Institute of Pharmacology, Polish Academy of Sciences, Department of Experimental Neuroendocrinology, PL 31-343 Krakow, Poland; rugmanim@if-pan.krakow.pl (R.M.);; 2 Jerzy Haber Institute of Catalysis and Surface Chemistry, Polish Academy of Sciences, PL 30-239 Krakow, Poland; tomasz.kruk@ikifp.edu.pl (T.K.); jacek.gurgul@ikifp.edu.pl (J.G.); krzysztof.szczepanowicz@ikifp.edu.pl (K.S.); marta.szczech@ikifp.edu.pl (M.S.); lilianna.szyk-warszynska@ikifp.edu.pl (L.S.-W.); piotr.warszynski@ikifp.edu.pl (P.W.)

**Keywords:** cerium oxide, neuroprotection, europium, nanoparticles

## Abstract

Cerium oxide nanoparticles (CeONPs) exhibiting antioxidant properties are investigated as potential tools for neurodegenerative diseases. Here, we synthesized polyacrylic acid conjugated cerium oxide (CeO) nanoparticles, and further to enhance their neuroprotective effect, Eu^3+^ was substituted at different concentrations (5, 10, 15 and 20 mol%) to the CeO, which can also impart fluorescence to the system. CeONPs and Eu-CeONPs in the size range of 15–30 nm were stable at room temperature. The X-ray Photoelectron Spectroscopy (XPS) analysis revealed the chemical state of Eu and Ce components, and we could conclude that all Eu^3+^ detected on the surface is well integrated into the cerium oxide lattice. The emission spectrum of Eu-CeO arising from the ^7^F_0_ → ^5^D_1_ MD and ^7^F_0_ → ^5^D_2_ transitions indicated the Eu^3+^ ion acting as a luminescence center. The fluorescence of Eu-CeONPs was visualized by depositing them at the surface of positively charged latex particles. The developed nanoparticles were safe for human neuronal-like cells. Compared with CeONPs, Eu-CeONPs at all concentrations exhibited enhanced neuroprotection against 6-OHDA, while the protection trend of Eu-CeO was similar to that of CeO against H_2_O_2_ in SH-SY5Y cells. Hence, the developed Eu-CeONPs could be further investigated as a potential theranostic probe.

## 1. Introduction

Nanotechnology is a promising approach for the theranostics (treatment and diagnosis) of neurodegenerative disorders, and several types of theranostic nanoparticles, both organic and inorganic nanoparticles, hybrid nanosystems including solid–lipid nanoparticles, polymeric nanoparticles, dendrimers, liposomes and metal oxide nanoparticles have been developed in the last decade [1,2]. Cerium oxide nanoparticles (CeONPs) are among the most promising theranostic agents, as they are biocompatible in vivo (IC_50_ above 3000 mg/kg) and possess unique redox properties [3]. The antioxidant activity of ceria is attributed to its ability to scavenge free radicals, such as superoxide and hydroxyl radicals, which is associated with the property of ceria to switch between Ce^4+^/Ce^3+^ oxidation states [4]. Nanoceria is currently being investigated for efficacy in several neurodegenerative disorders and has shown promising levels of neuroprotection [5]. CeONPs have been shown to mitigate neurodegenerative processes in depression and enhance neuronal plasticity [6]. Elshony et al. reported that CeONPs ameliorated the neurotoxicity induced by fipronil by scavenging reactive oxygen species (ROS) involving a decrease in malondialdehyde and nitric oxide, enhancing antioxidant enzyme activity as superoxide dismutase and glutathione peroxidase and normalizing the mRNA expression of brain function genes [7]. In our previous work, we reported the neuroprotective effects of polyacrylic acid conjugated cerium oxide (CeO-PAA) nanoparticles against neuronal cell damage induced by H_2_O_2_ and 6-OHDA oxidative stress inducers, which were associated with the inhibition of necrotic processes [8].

During the past decade, attempts have been made to control and enhance nanoceria’s redox and antioxidant properties [9,10]. Doping ceria with other elements, such as transition metals (Zr) or other lanthanides (La and Pr), has been proven to improve its redox properties due to the increased concentration of oxygen vacancies and oxygen mobility in ceria [11,12]. Ln^3+^-doped CeO_2_ nanomaterials were reported to enhance oxygen mobility, magnetism, catalytic reactivity and fluorescence compared to the CeO_2_ host [13]. Eu^3+^ ions are highly used as luminescence activators in red-emitting phosphors owing to their efficient f-f transitions [14]. A recent study reported Eu-doped ceria nanocrystals as nanoenzyme fluorescent probes for biosensing [15]. Hernández-Castillo et al. reported Eu-doped CeO_2_ as a potent antioxidant material for biological applications with antioxidant activity as a function of Eu^3+^ contents [16]. Enhancing the optical emission of cerium oxide nanoparticles is essential in biomedical applications, and this is achieved by studying the dependence on the oxygen ion vacancy and trivalent cerium, which, in turn, could be modified by Eu^3+^ dopant concentration [17]. The effects of EuCeO_2_ are linked to the particle’s ability to polarize microglia from a pro-inflammatory to an anti-inflammatory state, which facilitates cellular homeostasis, and they may serve as an immunomodulator for Alzheimer’s disease (AD) treatment [18]. As cited above, most reported articles describe the doping of trivalent ions in ceria for enhanced catalytic properties and antioxidant activities. Only a few studies, such as that of a co-doped Fe_3_O_4_ nanozyme, explored doping-enhanced neuroprotective potential [19].

In the present paper, we designed Eu^3+^-doped polyacrylic acid conjugated cerium oxide (Eu-CeO) nanoparticles to develop a theranostic agent that imparts luminescence and possesses neuroprotective activities. They were tested for their physiochemical properties and neuroprotective potential compared to those of CeONPs. Neuroprotection was tested in a commonly used human neuronal-like model of Parkinson’s disease utilizing retinoic acid-differentiated human neuroblastoma SH-SY5Y cells exposed to an oxidative stress inducer, hydrogen peroxide (H_2_O_2_), and dopaminergic neurotoxin, 6-hydroxydopamine (6-OHDA) [8].

## 2. Results and Discussion

### 2.1. Size Distribution and Stability

**Figure 1:**
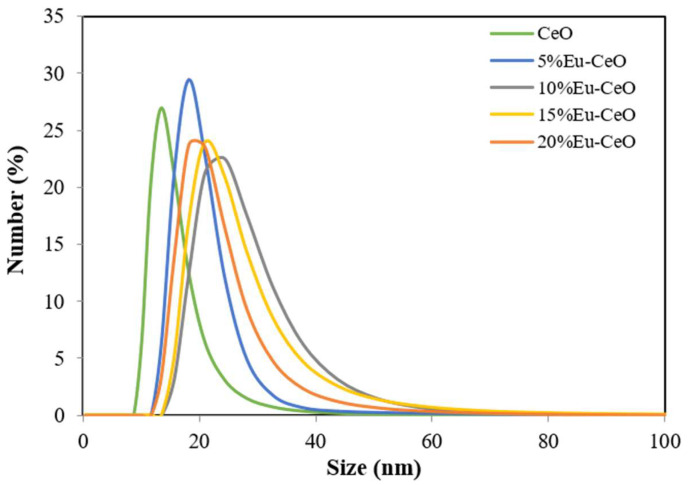
The size distributions for CeONPs and Eu-CeONPs at different dopant concentrations (5%, 10%, 15% and 20%) obtained by Dynamic Light Scattering (DLS).

CeO and Eu-CeO nanoparticles were synthesized using a low-temperature chemical precipitation method. The zeta potential of CeONPs was −54 ± 5 mV, whereas that of the Eu-CeONPs at different dopant concentrations (5%, 10%, 15% and 20%) varied from −57 to −61 ± 5 mV depending on the percentage of the dopant. The surface charge of nanoparticles ensured the electrostatic stabilization of their suspension, preventing the aggregation process. The size distributions for CeONPs and Eu-CeONPs are illustrated in Figure 1. The average size of nanoprobes was determined in the range of 15–30 nm. Eu^3+^ doping in the cerium matrix exhibited a slight increase in size, with the maximum peak (distribution by number) ranging from 18 to 24 nm, compared to 13.5 nm for CeONPs. However, the PDI values were maintained at 2.6, demonstrating uniformity of size and moderate monodispersity. Table 1 lists the average zeta potential values for CeO and Eu-CeO nanoparticles. The obtained suspensions of Eu-CeONPs were stable at room temperature for at least 2 months, and the zeta potential values and particle size distribution did not vary significantly over this period. These results are consistent with our previous data concerning CeO nanoparticles [8].

### 2.2. Absorption and Emission Characteristics

**Figure 2:**
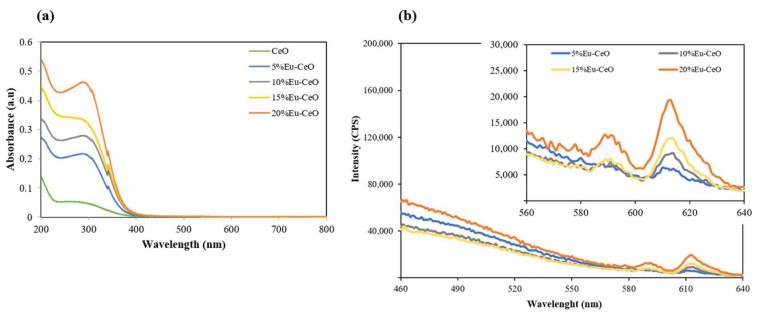
(**a**) UV–Vis absorption spectra of Eu^3+^-doped CeONPs as a function of doping concentration and (**b**) emission spectra of Eu-CeONPs (5%, 10%, 15% and 20% doping) recorded at an excitation wavelength of 380 nm.

Figure 2a depicts the absorption spectra of the CeONP suspension with different Eu^3+^ doping concentrations obtained in the UV–visible region. The spectra revealed a strong absorption band below 300 nm for CeO, which is due to the charge transfer from O^−2^ (2p) to Ce^4+^ (4f) orbitals in CeO_2_ [20]. A systematic increase in the peak intensity and its shift toward higher wavelengths as a function of Eu doping percentage can be attributed to the increase in the density of Eu atoms in the cerium matrix. The emission spectra for different concentrations of Eu^3+^-doped CeO nanoprobes, recorded at an excitation wavelength of 380 nm, are shown in Figure 2b. Characteristic emission with an intense and most prominent red emission (610 nm), which is credited to the ^7^F_0_ → ^5^D_2_ transition, can be observed in the luminescence spectra [21]. The luminescent transition between the 4f levels of rare earth ions is mainly due to the electric and magnetic dipole interactions [22], whereas the emission peak at 590 nm corresponds to the ^7^F_0_ → ^5^D_1_ transition of Eu^3+^ [23,24]. These results indicate that the Eu^3+^ ions act as luminescence centers in the CeO matrix.

### 2.3. Visualization of the Red Fluorescence of Eu-CeONPs

**Figure 3:**
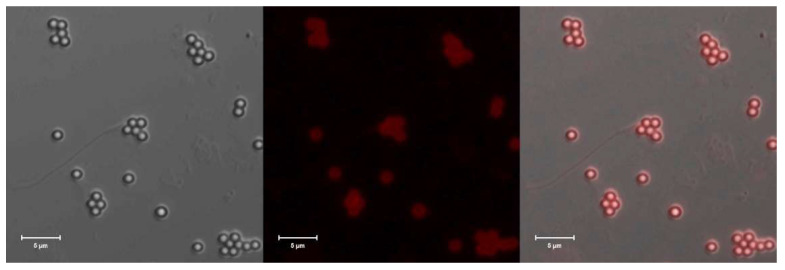
The visualization of the adsorbed Eu-CeONPs on polystyrene particles by confocal microscopy; (**left**) red transmitted light channel, (**middle**) red fluorescence channel and (**right**) channel superposition. The scale of the images corresponds to 5 µm.

The red fluorescence of Eu^3+^ (20%)-doped CeONPs was visualized using confocal microscopy. The Eu-CeONPs from the stock suspension were added to 1 cm^3^ of 0.1 wt% of a positively charged polystyrene latex microparticle solution (size 1 µm, zeta potential +55 mV, synthesized at ICSC PAS). The mixture was left for 4 h for Eu-CeONPs to adsorb on the surface of latex particles. Then, the zeta potential of the microparticles was measured. Its value of +20 mV confirmed the adsorption of Eu-CeONPs. Next, the channel slide with a glass bottom was filled with 100 mL of latex suspension with Eu-CeONPs. The positively charged particles adsorbed readily on a negatively charged glass surface and were visualized by Carl Zeiss LSM780 (Carl Zeiss, Jena, Germany) confocal microscopy. The fluorescence of Eu-CeONPs was excited with a 355 nm laser (30 mV), and the emission spectra were collected through the bandpass filters in the range of 610–660 nm. The polystyrene latex particles were simultaneously visualized through the PMT (transmitted light channel). The 63×/1.4 oil Plan Apochromat objective (Carl Zeiss, Jena, Germany) was used with optical zoom 2.4. The example of the image is illustrated in Figure 3. The red fluorescence of Eu-CeONPs can be seen in the fluorescence image, while the latex particles are clearly visible in the transparent light channel. The superposition of images confirms that Eu-CeONPs are adsorbed. These results indicate that the Eu^3+^-doped ceria nanoparticles can be easily visualized by fluorescent imaging, which opens a new perspective on the application of such nanosystems in theranostics.

### 2.4. X-ray Photoelectron Spectroscopy (XPS) Analysis

**Figure 4:**
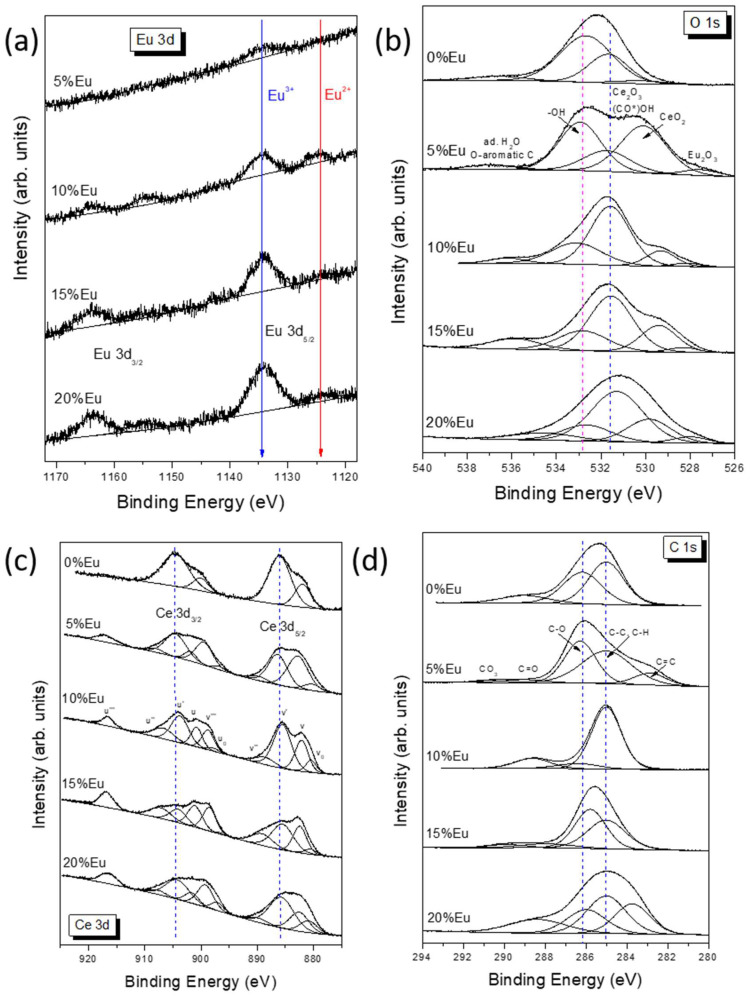
XPS: (**a**) 3d spectra of europium in xEu-CeO, (**b**) O 1s core excitation of xEu-CeO, (**c**) 3d spectra of cerium in xEu-CeO and (**d**) C 1s core excitation of xEu-CeO. Dash lines are guide to the eye only.

XPS measurements were performed to clarify changes in the electronic structure of -CeO nanoparticles with different amounts of europium. The high-resolution Eu 3d spectra (Figure 4a) show well-separated Eu 3d5/2 peaks at 1134.3 eV and 1124.2 eV, which can be attributed to Eu(III) and the shake-off satellites of Eu 3d, respectively [25,26,27]. Later peaks can also be attributed to Eu(II) present on the surface, which is possible when Eu(III) is reduced to Eu(II) when oxygen defects are created. The spin-orbit splitting is 29.5 eV, in agreement with the literature [28]. The components corresponding to Eu(III) occur at the same BE value of 1134.3 eV, regardless of the amount of Eu, while the Eu(II) component changes position slightly, which may indicate various degrees of oxide defectiveness. The 5%Eu sample does not contain Eu (II) species at all, while the highest Eu(II)/Eu(III) ratio is observed in the 10%Eu sample and is close to 0.54. Samples with a high europium content are characterized by a small contribution of Eu(II) in the spectrum, at around 15%. XPS measurements of O 1s signals (Figure 4b) were made to interpret the chemical states of oxygen on the surface of Eu-CeO samples. The deconvoluted spectra show the presence of five components which can be attributed to (i) Eu-O bonds (527.5–528.3 eV), (ii) CeO_2_ (529.3–530.2 eV), (iii) Ce_2_O_3_ and carboxyl groups (531.3–531.7 eV), (iv) OH- groups (532.7–533.1 eV) and (v) adsorbed water and oxygen-aromatic carbon bonds (>534.5 eV). The relative contribution of the components is presented in Table 2.

The results of the study conducted on Eu_2_O_3_ thin films obtained by Kumar et al. [26] showed the presence of two O 1s peaks at 528.9 and 531.1 eV. The most intense peak centered at 528.9 eV was assigned to the Eu–O bonds in Eu_2_O_3_, whereas the peak located at higher binding energy was attributed to the OH^−^ groups adsorbed at the sample surface. It is worth noting that a different interpretation of oxygen contributions was proposed by Kang et al. [28] for the characterization of Eu(OH)_3_ and Eu_2_O_3_ nanorods. Two peaks found at a binding energy of 528.9 and 530.9 eV in Eu(OH)_3_, as well as 529.2 and 531.3 eV in Eu_2_O_3,_ were attributed to surface oxygen vacancies and lattice oxygen, respectively. Thus, it is possible that europium-containing bonds can also participate in component (iii). In pure CeO_2_ nanoparticles, only one peak at 528.7 eV was identified as the lattice oxygen of the host [29]. Also, Mullins et al. reported a single O 1s peak at 530.4 eV in an oxidized thin film of CeO_2_(001) [30]. In contrast, the O 1s spectrum of thin films of CeO_2_ grown on Si substrates consisted of a sharp peak at 529.4 eV and a widened feature at 531.7 eV associated with hydroxyl groups [31]. The Xe ion irradiation showed a reduction of some CeO_2_ to Ce_2_O_3,_ which resulted in a shift of the main oxygen line by 0.8 toward higher binding energies.

A detailed analysis of the oxygen spectra shows that the least component corresponding to CeO_2_ was observed in the 0%-Eu sample and the most in the 5%-Eu sample. It is worth recalling that no Eu^2+^ component was observed in the 5%-Eu sample. Moreover, an increase in the amount of Eu resulted in the appearance of the Eu^2+^ component and an increase in the contribution of the Ce^3+^ component. Most likely, the doping of Eu ions in the CeO_2_ creates oxygen vacancies, which partially transform Ce^4+^ species into Ce^3+^ ones.

The Ce 3d XPS spectra of xEu-CeO are presented in Figure 4c. Due to the complex nature of spectra, it was necessary to apply a model that considered a multiplicity of 3d states originating from different Ce 4f level occupancies. The 10-component approach was chosen according to the articles of Paparazzo and Leel et al. [32,33]. The Ce 3d_5/2_ and Ce 3d_3/2_ multiplets are labeled ‘v’ and ‘u’, respectively. The peaks at 882.5 (v), 889.7 (v″) and 899.2 eV (v′′′), as well as at 901.6 (u), 907.7 (u″) and 916.5 eV (u′′′), were identified as being derived from Ce (IV) species, whereas 880.9 (v_0_), 885.7 (v′), 897.1 (u_0_) and 904.2 eV (u′) were identified as being derived from Ce(III) ones. The location of the u′′′ line considered as a reference for Ce(IV) was in perfect agreement with the literature [32]. The surface concentration of Ce(III) was estimated from the Ce(III)/(Ce(III) + Ce(IV)) peak area ratio [25,32]. As illustrated in Table 3, the 0% sample has the highest Ce(III) content (over 77%). In samples 5%-Eu, 10%-Eu and 20%-Eu, the Ce(III)/Ce(IV) ratio is in the range of 0.8–1.3, while the lowest contribution of Ce(III) (about 33%) is observed in sample 15%-Eu. The presence of trivalent Ce species can be related to Ce_2_O_3_ or CeO_2_ with oxygen vacancies.

Several contributions can be distinguished in the C 1s spectra (Figure 4d). These components can be assigned as follows (Table 4): (i) carbides C=C (282.3–283.7 eV), (ii) organic contaminants C-C/C-H (285.0 eV), (iii) C-O groups (285.8–286.4 eV), (iv) carbonyl C=O groups (287.9 eV), (v) carboxyl O-C=O groups (288.4–289.0 eV) and (vi) carbonates (289.9–290.7 eV) [34]. The hydrocarbon contamination was used as an internal calibration for all samples (C-H peak at 285.0 eV). It is worth noting that the above-mentioned assignment of chemical bonds to individual components may not be quantitatively perfect because a Ce 4s line can also contribute to the C 1s spectra near BE = 289 eV [31].

### 2.5. Biosafety Evaluation of Eu-CeO Nanoparticles

**Figure 5:**
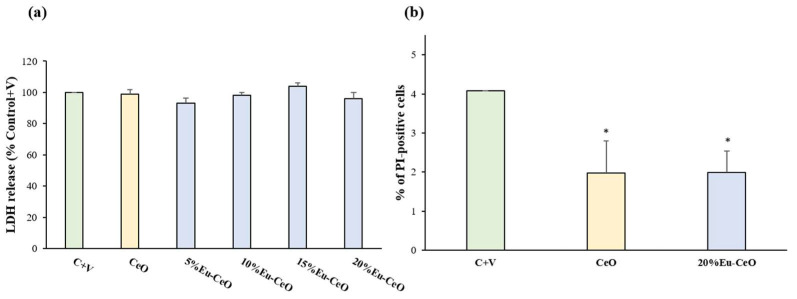
Biosafety of CeONPs and Eu-CeONPs (5, 10, 15 and 20%). The RA-SH-SY5Y cells were treated with CeONPs and Eu-CeONPs for 24 h. (**a**) Data from LDH release assay normalized to the vehicle-treated cells and (**b**) data from flow cytometry analysis of propidium iodide-stained RA-SH-SY5Y cells shown as a percentage of PI-positive cells. The data were analyzed by one-way ANOVA followed by Duncan’s post hoc test and are presented as the mean ± SEM. * *p* < 0.05 versus vehicle-treated cells; C—control; V—vehicle.

Twenty-four hours of treatment with CeO and Eu-CeO nanoparticles with varying Eu^3+^ content (5%, 10%, 15% and 20%) did not evoke any detrimental effect on RA-SH-SY5Y cells in comparison with vehicle-treated cells as was measured using a cytotoxicity (LDH release) assay. This was further evidenced by propidium iodide (PI) staining of cells treated for 24 h with CeONPs and 20%-Eu-CeONPs and flow cytometry analysis, where no significant increase was observed in the number of PI-positive cells when compared to the vehicle-treated group (Figure 5b). Interestingly, we found that cells treated with CeONPs and 20%-Eu-CeONPs exhibited a lower number of PI-positive cells when compared to control cells (Figure 5b). These data confirm our previous findings, showing that PAA-CeO (0.03 M) nanoparticles are safe for undifferentiated- and retinoic acid-differentiated SH-SY5Y cells [8] and extend these data to Eu-CeONPs, evidencing additional protective potency of these NPs under basal conditions (cell cultured in medium with low serum content).

### 2.6. Neuroprotective Effects of Eu-CeO Nanoparticles against the H_2_O_2_ and 6-OHDA-Induced Cell Damage

**Figure 6:**
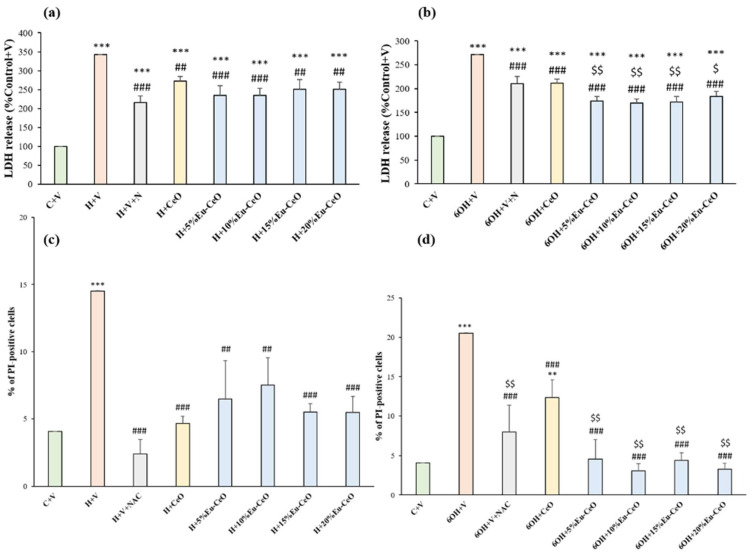
Neuroprotective effects of CeO and Eu-CeO nanoparticles in RA-SH-SY5Y cells. The cells were pretreated for 30 min with nanoparticles, followed by 24 h of treatment with H_2_O_2_ (0.5 mM) and 6-OHDA (0.2 mM). An antioxidant N-acetyl cysteine (NAC, 1 mM) was used as a positive control. The cytotoxicity evoked by (**a**) H_2_O_2_ and (**b**) 6-OHDA in RA-SH-SY5Y was measured using an LDH release assay. The data were normalized to the vehicle-treated cells, analyzed by one-way ANOVA followed by Duncan’s post hoc test and are presented as the mean ± SEM from 4–9 independent experiments. Flow cytometry analysis of propidium iodide (PI)-stained cells against (**c**) H_2_O_2_ and (**d**) 6-OHDA-evoked damage. Data from 2–3 independent experiments with 2 repetitions each were analyzed by one-way ANOVA followed by Duncan’s post hoc test and are presented as the mean ± SEM of PI-positive cells. ** *p* < 0.01 and *** *p* < 0.001 versus vehicle-treated cells; ## *p* < 0.01 and ### *p* < 0.001 versus H_2_O_2_/6-OHDA-treated cells; $ *p* < 0.05 and $$ *p* < 0.01 versus 6-OHDA+CeO. C—control, H—H_2_O_2_, 6OH—6-hydroxydopamine, N—NAC, V—vehicle.

In order to test the neuroprotective potential of CeONPs and to understand the influence of Eu^3+^ doping in CeONPs, the RA-SH-SY5Y cells were pretreated with developed nanoparticles for 30 min followed by 24 h exposure to cell-damaging factors (H_2_O_2_ and 6-OHDA). We observed approximately a 3.5-fold increase in cytotoxicity after the incubation of RA-SH-SY5Y cells with H_2_O_2_ (0.5 mM) compared to vehicle-treated cells, which was substantially reduced by all tested nanoprobes (Figure 6a). In the H_2_O_2_ model, Eu-CeONPs (5–20%) exhibited protection similar to CeONPs, which was comparable to neuroprotection mediated by positive control, NAC. In the model of 6-OHDA-induced cell damage in RA-SH-SY5Y, we observed an over 2.7-fold increase in LDH release after exposure to 0.2 mM 6-OHDA, which was substantially reduced by CeONPs, Eu-CeONPs (5–20%) and NAC (Figure 6b). In the 6-OHDA model, we found a significantly higher reduction in LDH release by Eu-CeONPs at all doping concentrations compared to the effect of CeONPs (Figure 6b).

**Figure 7:**
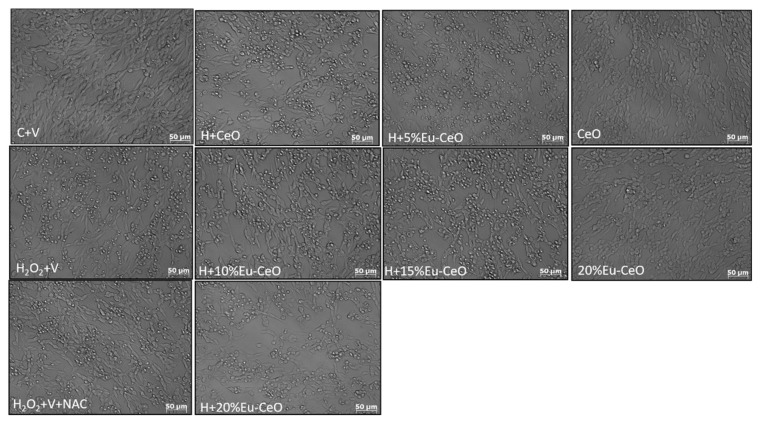
Representative DIC (differential interference contrast) microphotographs of RA-SHSY5Y cells pretreated for 30 min with CeO and Eu-CeO (5–20%) followed by 24 h of incubation with H_2_O_2_ (0.5 mM). An antioxidant N-acetyl-cysteine (NAC, 1 mM) was used as the positive control in this oxidative stress model.

**Figure 8:**
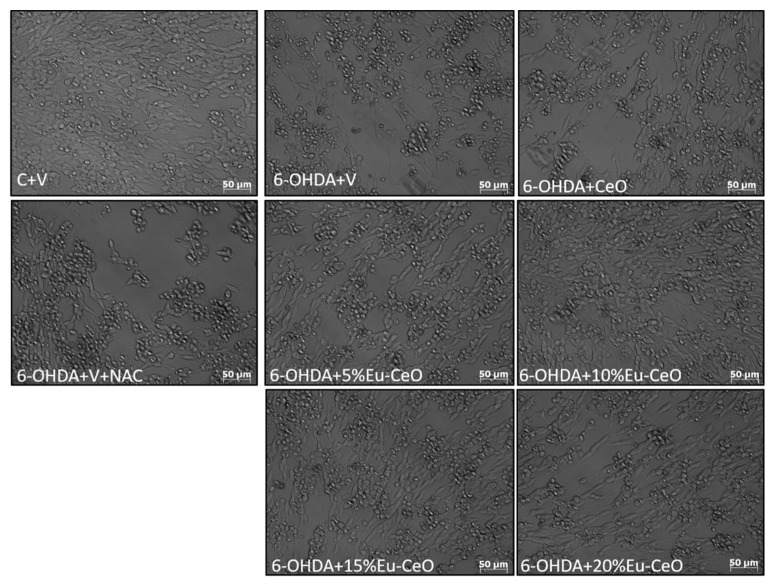
Representative DIC (differential interference contrast) microphotographs of RA-SH-SY5Y cells pretreated for 30 min with CeO and Eu-CeO (5–20%) followed by 24 h of incubation with 6-OHDA (0.2 mM). An antioxidant N-acetyl-cysteine (NAC, 1 mM) was used as the positive control in this oxidative stress model.

Further, the neuroprotective effects were confirmed by PI staining in RA-SH-SY5Y cells, which was consistent with the LDH release assay. We found that both Eu-CeONPs and CeONPs at various concentrations in a similar extent attenuated the number of damaged (PI-positive) nuclei induced by H_2_O_2_ (Figure 6c). In the 6-OHDA model, Eu-CeONPs (5–20%) showed significantly higher protection than CeONPs (Figure 6d). Moreover, protection mediated by nanoparticles in both cell damage models in RA-SH-SY5Y cells was also evidenced by light microscopy DIC imaging (Figure 7 and Figure 8). The above data obtained with CeONPs correlate well with our previous findings from undifferentiated and RA-differentiated cells, where we observed significant neuroprotection by these nanoparticles against the cell damage evoked by oxidative stress inducers [8].

### 2.7. Effect of Eu-CeO Nanoparticles on the H_2_O_2_-Induced Increase in Intracellular Reactive Oxygen Species (ROS) Level

In our previous study, we showed that the neuroprotective effects of CeONPs (0.03 M) against the H_2_O_2_-evoked cell damage in undifferentiated SH-SY5Y cells were not associated with the direct inhibition of intracellular ROS production [8]. Since it has been suggested from a chemical structure perspective that the Eu-doping of CeO_2_ nanoparticles could increase its antioxidant properties [9], we verified this hypothesis in our cellular system. Not only did we not observe any attenuation by Eu-CeO (5–20%) or CeO nanoparticles of the H_2_O_2_-induced ROS production, but we even found a significant exaggeration of this parameter by all tested nanoprobes. However, CeONPs and Eu-CeONPs (5–20%) alone did not evoke a significant change in CM-DCF fluorescence compared to vehicle-treated cells (Table 5). The above findings confirm our previous observations that the direct inhibition of ROS by CeO is not associated with its protection against the H_2_O_2_-evoked cell and extend them also to the Eu-doped CeO system.

### 2.8. Effect of Eu-CeONPs on Caspase-3 Activity

Based on our previous studies, which showed partial attenuation of apoptosis measured by caspase-3 activation in the mechanism of 6-OHDA-evoked cell damage [8], we tested the effect of Eu-CeONPs on this apoptotic cell death marker. We observed the activation of caspase-3 after 18 h of treatment with 6-OHDA in RA-SH-SY5Y cells, which was significantly attenuated by Eu-CeONPs in all doping concentrations (5–20%) but did not find a significant impact of CeONPs (Table 6). These data evidence that Eu doping could enhance the inhibitory effect of CeO on caspase-3 activity, which could contribute to better protection mediated by EuCeONPs in the 6-OHDA model of cell damage.

In this study, we demonstrated for the first time that doping CeO with Eu did not change the protection range mediated by the parental nanoprobe as observed in the H_2_O_2_ model of cell damage, and in the case of the 6-OHDA model, we even observed enhanced protection. It should be noted that in the field of neuroprotection, there is limited data on the effectiveness of the doping of CeO nanoparticles with Eu^3+^. In a recent report, it was demonstrated that EuCeO_2_ nanoparticles synthesized through the solvothermal method attenuated microglia BV2 inflammatory activities after Aβ1-42 exposure by increasing the cells’ phagocytic and Aβ degradation activities [18]. However, in that study, there was no direct comparison to the effectiveness of CeO_2_ nanoparticles since only the lipid nanoparticles (LNPs) without cerium oxide were used as control. In our opinion, in comparison with native CeONPs, the developed Eu-CeONP seems to be a promising theranostic agent for Parkinson’s disease monitoring and treatment.

## 3. Materials and Methods

### 3.1. Materials

Cerium (III) nitrate hexahydrate, ammonium cerium (IV) nitrate, europium (III) nitrate hydrate, polyacrylic acid sodium salt (Mw = 5100) and ammonium hydroxide solution (30% NH_3_ in H_2_O) were procured from Sigma-Aldrich (Sigma-Aldrich Chemie GmbH, Taufkirchen, Germany). For cell culture, Dulbecco’s Modified Eagle Medium (DMEM), Trypsin/EDTA (0.25%) solution, heat-inactivated fetal bovine serum (FBS), Dulbecco’s phosphate-buffered saline (DPBS, without calcium and magnesium), penicillin and streptomycin mixture and FluoroBrite™ DMEM were procured from Gibco (Invitrogen, Paisley, UK). The cytotoxicity detection kit (LDH release assay) was from Roche Diagnostic (Mannheim, Germany). Ac-DEVD-AMC, a caspase-3 fluorogenic substrate, was obtained from Enzo Life Sciences (New York, NY, USA). CM-H2DCFDA assay was purchased from Molecular Probes (Life Technologies Corporation, Eugene, OR, USA). Triton X-100, N-acetyl cysteine (NAC), propidium iodide, stabilized hydrogen peroxide solution (30% H_2_O_2_) and 6-hydroxydopamine hydrochloride were sourced from Sigma Aldrich (Sigma-Aldrich Chemie GmbH, Taufkirchen, Germany).

### 3.2. Synthesis and Characterization of Eu-CeO Nanoparticles

The PAA-stabilized CeO_2_ nanoparticles were synthesized using the low-temperature precipitation method. A mixed solution containing 30 mM cerium(III) nitrate hexahydrate salt and ammonium cerium(IV) nitrate salt with 10% by weight of PAA (MW 5100) was prepared, and 30% ammonium hydroxide solution was added to this in a dropwise manner. Eu-doped cerium nanoparticles were synthesized similarly. During the synthesis of Eu-doped CeO nanoparticles, the concentration of ammonium cerium(IV) nitrate salt ((NH_4_)_2_[Ce(NO_3_)_6_]) remained constant and amounted to 30 mM, whereas the concentration of cerium(III) nitrate hexahydrate salt (Ce(NO_3_)_3_·6H_2_O) was changed depending on the degree of doping. The amount of europium(III) nitrate hydrate salt (Eu(NO_3_)_3_·5H_2_O) was added so that the total concentration of cerium nitrate hexahydrate and europium nitrate hydrate salts was 30 mM. For example, for 20% Eu doping, 0.006 M Eu salt and 0.024M Ce salt were used and similarly for other levels of Eu doping. Several syntheses of Eu-CeONPs with 5%, 10%, 15% and 20% doping were conducted to compare properties and select the most optimal suspensions. After continuous stirring for 24 h, the obtained suspensions of doped Eu-CeO nanoparticles were dialyzed against 5L of distilled water at pH 7 for 2 days. The water was changed 3 times a day.

The hydrodynamic particle size and zeta potential of Eu-CeONPs were determined by Dynamic Light Scattering (DLS) (Zetasizer Nano Series, Malvern Instruments, Malvern, Worcestershire, UK). The sample was measured at 25 °C in triplicate with at least 20 measurements using water as dispersant with parameters set for cerium oxide (refractive index = 2.2 and absorption = 0.001). The zeta potential measurements were conducted using LDE (Laser Doppler Electrophoresis) and Zetasizer Nano Series (Malvern Instruments, Malvern, Worcestershire, UK). The X-ray Photoelectron Spectroscopy (XPS) measurements were carried out in a multi-chamber UHV system equipped with a hemispherical analyzer (SES R4000, Gammadata Scienta, Uppsala, Sweden), and the numerical analysis was performed with CasaXPS 2.3.23 software after subtracting the Shirley-type background. The experimental results were fitted using a profile with a variable ratio (70:30) of Gaussian and Lorentzian lines [35]. The absorption spectra were recorded with a UV-Vis spectrometer (Shimadzu Corporation, Duisburg, Germany) in the range of 200–800 nm. Photoluminescence spectra of Eu-CeONPs were recorded using a Fluorolog^®^-3 spectrofluorometer (HORIBA Jobin Yvon, Longjumeau, France) at the excitation wavelength of 380 nm and emission spectra with the scan range from 400 to 660 nm with 5 nm slit widths and an integration time of 0.1 s. All samples were characterized as synthesized. To confirm the imaging abilities of synthesized nanoparticles by optical modalities, the nanoprobes were deposited on the surface of positively charged latex microparticles and visualized by Carl Zeiss LSM780 (Carl Zeiss, Jena, Germany) confocal microscopy.

### 3.3. Cell Culture

Human neuroblastoma SH-SY5Y cells (ATCC CRL-2266, Manassas, VA, USA) were cultured in DMEM supplemented with a 10% *v*/*v* FBS and 1% *v*/*v* penicillin/streptomycin mixture, as described previously [36]. After enzymatic detachment from a surface (0.05% Trypsin/EDTA), the cells were manually counted and seeded at the density of 2 × 10^4^, 1 × 10^5^ and 5 × 10^5^ cells per well into 96-, 24- and 6-well plates, respectively, in cell culture medium supplemented with 10 μM retinoic acid (RA). The cells were differentiated for 6 days with the culture medium exchange every 2 days. On the sixth day of cell culture, the culture medium was exchanged with an experimental medium containing DMEM, 1% *v*/*v* penicillin/streptomycin mixture and 1% *v*/*v* FBS.

### 3.4. Cell Treatment

First, RA-SH-SY5Y cells were treated with 10% *v*/*v* of CeONPs (0.03 M) and Eu-CeONPs (5, 10, 15 and 20%) for 24 h to assess the biosafety of the nanoparticles. Next, to test the neuroprotective potential of developed nanoprobes, the cells were pretreated with 10% *v*/*v* nanoparticles or vehicle (sterile distilled water) for 30 min followed by 24 h exposure to H_2_O_2_ (IC_50_ of 0.5 mM) or 6-OHDA (IC_50_ 0.2 mM). The concentrations of H_2_O_2_ and 6-OHDA chosen to induce significant cell damage in neuronally differentiated SH-SY5Y cells were optimized in our previous work [8,36,37]. As a positive control for both cell-damaging factors, an antioxidant N-acetyl cysteine (NAC, mM) was employed, which was given concomitantly with oxidative stress inducers.

### 3.5. Cytotoxicity Assay

The lactate dehydrogenase (LDH) level released from damaged cells into culture media, a cell death marker, was measured using Cytotoxicity Detection Kit (Roche, Mannheim, Germany) as described previously [8]. The absorbance of each sample was measured with a multi-well-plate reader (Infinite^®^ M200 PRO, Tecan Austria GmbH, Grodig, Austria) at 490 nm. The data after normalization to the vehicle-treated cells (100%) are presented as a mean ± SEM established from 4–9 independent experiments with 3–5 replicates each.

### 3.6. Microscopic Assessment of Morphological Changes

For confirmation of biochemical results on protection mediated by Eu-CeONPs in RA-SH-SY5Y cells, 24 h after treatment, the cells were placed in FluoroBrite™ DMEM and were visualized using inverted fluorescence microscope (AxioObserver, Carl Zeiss, Jena, Germany). The microscopic evaluation was carried out using the DIC (differential interference contrast) technique, and microphotographs were taken using a black–white camera (Axio-CamMRm, Carl Zeiss).

### 3.7. Propidium Iodide Staining and Flow Cytometry

Propidium iodide staining of RA-SH-SY5Y cells, treated with Eu-CeONPs in a 24-well plate, was performed to verify the results from the LDH release assay. Briefly, twenty-four hours after cell treatment, they were collected on ice and stained with propidium iodide (PI, 10 µg/mL in DPBS), as reported earlier [6]. Cells treated with Triton X-100 for 5 min were used as a positive control to acquire a maximal signal in PI staining. The cells (1 × 10^4^) were analyzed in the fluorescence channel for PerCP-Cy5-5-A (red fluorescence) using BD FACS Canto II System and BD FACSDiva™ v5.0.1 Software (BD Biosciences, San Jose, CA, USA). The PI-positive cells (exhibiting loss of cell membrane integrity) represent necrotic and late apoptotic cells. Data are presented as a percentage of PI-positive cells (±SEM) established from 3 independent experiments with 2 replicates each.

### 3.8. Measurement of Intracellular Reactive Oxygen Species (ROS)

The CM-H2DCFDA (5-(and-6)-chloromethyl-2′,7′-dichlorodihydrofluorescein diacetate, acetyl ester) probe was used for estimation of the intracellular ROS level according to procedure described in detail in our previous work [8]. The RA-SH-SY5Y cells growing and differentiated in 24-well format were loaded with 5 μM CM-H2DCFDA dissolved in FluoroBrite™ DMEM and placed in the incubator. After 5 min of incubation, the cells were treated with 10% *v*/*v* of CeONPs (0.03 M) and Eu-CeONPs (5, 10, 15 and 20%) for 25 min followed by 30 min exposure to H_2_O_2_ (1 mM). Next, the cells were washed twice with pre-warmed FluoroBrite™ DMEM, collected and centrifuged in 1.5 mL tubes. The cell pellet was placed in 200 μL of cold DPBS without calcium and magnesium, and 1 × 10^4^ cells were analyzed using BD FACS Canto II System and BD FACSDiva™ v5.0.1 Software (BD Biosciences, San Jose, CA, USA) in the fluorescence channel for FITC (green fluorescence). Mean FITC fluorescence intensity was recorded for each sample. Data were normalized to vehicle-treated cells (100%) and are presented as a mean ± SEM established from 3 independent experiments with 2 replicates each.

### 3.9. Caspase-3 Activity Assay

To evaluate the influence of Eu-CeONPs on apoptotic changes induced by 6-OHDA, a caspase-3 activity assay was performed, as described earlier [8]. The RA-SH-SY5Y cells were grown in a 6-well plate and were pretreated for 30 min with Eu-CeONPs or caspase-3 inhibitor, Ac-DEVD-CHO (20 µM), followed by 18 h exposure to 6-OHDA. Data were calculated as a percent of vehicle-treated cells and are presented as the mean ± SEM from 4 independent experiments with 2 replicates each. Our previous study reveals that CeONPs at 0.03 M did not affect the H_2_O_2_-induced caspase-3 activity; hence, the measurements were not attempted with Eu-CeONPs [8].

### 3.10. Statistical Analysis

The data were analyzed with Statistica 13.3 software (StatSoft Inc., Tulsa, OK, USA). One-way analysis of variance (ANOVA) with post hoc Duncan’s test for multiple comparisons with assumed *p* < 0.05 was used.

## 4. Conclusions

The Eu-CeO nanoprobes were successfully synthesized by doping varying concentrations of Eu^3+^ (5, 10, 15 and 20%) in polyacrylic acid conjugated cerium oxide through a chemical precipitation technique. Eu-CeONPs in the size range of 15–30 nm imparted fluorescence and enhanced neuroprotection to the parent nanoprobes, which reveals the potential of the developed nanoparticles as a theranostic agent. The structural investigation through XPS indicated the existence of Eu^3+^ and Eu^2+^ ions in the CeO matrix, and Eu^3+^ ions acting as luminescence centers were confirmed using a spectrofluorometer. Eu-CeONPs were safe for retinoic RA-SH-SY5Y cells and exhibited enhanced protection against 6-OHDA in all concentrations compared with native CeONPs. Moreover, our study also points out that the mechanisms of neuroprotection in both H_2_O_2_ and 6-OHDA models could be associated with the inhibition of necrotic processes, but a role of the attenuation of activity of executor apoptotic protease, caspase-3 (6-OHDA model), cannot be excluded. Thus, the potential clinical utility of this type of nanoparticle should be further evaluated in animal models of Parkinson’s disease or other neurodegenerative diseases.

## Figures and Tables

**Table 1:** The zeta potential of CeO and Eu-CeO at different dopant concentrations (5%, 10%, 15% and 20%). The error of the zeta potential measurements ±5 mV.

Sample	Zeta Potential (mV)
CeO	−54
5%Eu-CeO	−58
10%Eu-CeO	−59
15%Eu-CeO	−59
20%Eu-CeO	−61

**Table 2:** Binding energy (eV) and percentage of components (%) of O 1s core excitation in xEu-CeO samples.

Sample	Eu-O	CeO_2_	Ce_2_O_3_, O-C=O	-OH	Ad. H_2_O O-Aromatic C
CeO	---	530.2 (3.5)	531.7 (28.9)	532.7 (62.0)	536.5 (5.6)
5%Eu-CeO	527.5 (3.6)	530.1 (39.0)	531.7 (17.1)	532.9 (36.3)	536.9 (4.0)
10%Eu-CeO	528.1 (2.1)	529.3 (10.8)	531.6 (56.4)	533.1 (26.2)	536.2 (4.5)
15%Eu-CeO	528.3 (7.4)	529.4 (18.3)	531.5 (45.6)	532.8 (18.6)	535.9 (10.1)
20%Eu-CeO	528.0 (4.1)	529.8 (19.8)	531.3 (51.6)	532.7 (15.6)	534.5 (8.9)

**Table 3:** Binding energies (eV) of various Ce 3d XPS components of xEu-CeO nanoprobe.

Sample	Ce 3d_5/2_ (eV)					Ce 3d_3/2_ (eV)					Ce^3+^ (%)	Ce^4+^ (%)	Ce^3+^/Ce^4+^
	v_0_	v	v′	v″	v′′′	u_0_	u	u′	u″	u′′′			
CeO	-	881.9	886.0	-	-	-	900.1	904.5	-	917.1	77.2	22.8	3.39
5%Eu-CeO	880.2	882.8	886.4	889.7	899.5	896.6	901.9	904.5	908.0	916.9	45.6	54.4	0.84
10%Eu-CeO	880.4	882.1	885.5	888.6	898.8	897.9	900.8	903.8	906.7	916.6	51.1	48.9	1.04
15%Eu-CeO	880.5	882.4	885.5	889.1	898.5	896.6	901.1	904.1	907.2	916.8	33.5	66.5	0.50
20%Eu-CeO	880.9	882.5	885.7	889.7	899.2	897.1	901.6	904.2	907.7	916.5	56.2	43.8	1.28

**Table 4:** Binding energy (eV) and percentage of components (%) of C 1s core excitation in xEu-CeO.

Sample	C=C	C-C	C-O	C=O	O-C=O	CO_3_
5%Eu-CeO	282.8 (10.9)	285.0 (46.7)	286.3 (37.5)	---	289.0 (3.3)	290.7 (1.6)
10%Eu-CeO	282.9 (0.6)	285.0 (75.7)	286.4 (8.9)	---	288.6 (14.8)	---
15%Eu-CeO	282.3 (0.3)	285.0 (41.4)	285.8 (45.9)	287.9 (7.8)	---	289.9 (4.6)
20%Eu-CeO	283.7 (24.8)	285.0 (34.9)	286.0 (22.0)	---	288.4 (18.3)	---

**Table 5:** Effect of CeO and 5–20% Eu-CeO on the H_2_O_2_-induced increase in intracellular reactive oxygen species (ROS) level.

Sample	CM-DCF Intensity (% Control + V)
Control + Vehicle	100.00 ± 0.00
H + V	272.01 ± 3.74 ***^, ###^
H + CeO	356.59 ± 11.35 ***^, ###^
H + 5%Eu-CeO	368.61 ± 12.05 ***^, ###^
H + 10%Eu-CeO	385.44 ± 14.16 ***^, ###^
H + 15%Eu-CeO	364.25 ± 20.63 ***^, ###^
H + 20%Eu-CeO	403.54 ± 24.49 ***^, ###^
CeO	109.62 ± 5.62
5%Eu-CeO	105.69 ± 2.27
10%Eu-CeO	102.89 ± 5.44
15%Eu-CeO	105.85 ± 8.03
20%Eu-CeO	104.70 ± 1.16

The RA-SH-SY5Y cells were loaded with 5 μM of CM-H2DFFDA, incubated with 10% *v*/*v* CeO (0.03 M) and Eu-CeONPs (5, 10, 15 and 20%) for 25 min, followed by 30 min exposure to 1 mM H_2_O_2_. The mean CM-DCF fluorescence intensity was measured in 1 × 10^4^ cells by flow cytometry (in the FITC panel). The data were normalized to vehicle-treated cells (100%) and are presented as a mean ± SEM from 3 independent experiments with 2 replicates each. *** *p* < 0.001 vs. vehicle-treated cells; ^###^ *p* < 0.001 vs. H_2_O_2_-treated cells.

**Table 6:** Effects of CeONPs and Eu-CeONPs on the 6-OHDA-induced caspase-3 activity.

Sample	Caspase-3 Activity (%Control + V)	*p*-Value vs. 6OHDA from Duncan’s Post Hoc Test
Control + Vehicle	100.00 ± 0.00	
6-OHDA + Vehicle	499.23 ± 0.96 ***	
6-OHDA + CeO	428.56 ± 33.80 ***	0.113611
6-OHDA + 5%Eu-CeO	371.69 ± 32.54 ***^, ##^	0.007474
6-OHDA + 10%Eu-CeO	332.69 ± 42.39 ***^, ##^	0.001045
6-OHDA + 15%Eu-CeO	353.96 ± 36.46 ***^, ##^	0.002326
6-OHDA + 20%Eu-CeO	343.03 ± 37.43 ***^, ##^	0.001793

The RA-SH-SY5Y cells were pretreated for 30 min with CeONPs and Eu-CeONPs, followed by 18 h of treatment with 6-OHDA (0.2 mM). Data were normalized to vehicle-treated cells (control) and are presented as the mean ± SEM from four separate experiments with two repetitions each. *** *p* < 0.001 vs. vehicle-treated cells; ^##^ *p* < 0.01 vs. 6-OHDA-treated cells.

## Data Availability

The data presented in this study are available on request from the corresponding author.
